# Coping in Teams: Exploring Athletes’ Communal Coping Strategies to Deal With Shared Stressors

**DOI:** 10.3389/fpsyg.2018.01908

**Published:** 2018-10-09

**Authors:** Chloé Leprince, Fabienne D’Arripe-Longueville, Julie Doron

**Affiliations:** ^1^Institut National du Sport, de l’Expertise et de la Performance, Paris, France; ^2^LAMHESS (EA6312), University of Côte d’Azur, Nice, France; ^3^Laboratory Movement, Interactions, Performance (EA 4334), Faculty of Sport Sciences, University of Nantes, Nantes, France

**Keywords:** shared stressors, communal coping, team sports, interpersonal perspective, team performance

## Abstract

Although sport psychology research has mainly focused on stress and coping as intrapersonal processes, stressful circumstances are often experienced in social groups and coping emerges as a combination of individual and group effort (Tamminen and Gaudreau, 2014). Based on Lyons et al. (1998) model of communal coping, this study aimed to address the lack of knowledge about stress and coping as an interpersonal process, by exploring shared stressors and communal coping strategies within team sports. Semi-structured interviews were conducted with 10 team sport athletes (seven males, three females; *M*_age_ = 26.3 years, *SD*_age_ = 7.67, range 15–38) who participated in different team sports (football, rugby, volleyball, ice hockey, and basketball). Data were analyzed using an inductive thematic analytic procedure. The results revealed four themes of shared stressors involving issues relating to social pressure, relationships between teammates, performance, or logistics and organization; and four themes of communal coping, namely: problem-focused communal efforts, relationship-focused coping, communal management of emotions, and communal goal withdrawal. The results provided empirical support to the communal coping model (Lyons et al., 1998) and extend understanding of coping processes as defined initially by the CMRT of emotion (Lazarus, 1999, 2000a). This study provided unique insight into the nature of communal coping in sport and performance setting, and specifically, how stressors are apprehended in team sports and how athletes can collaborate to deal with shared stressors during competitive encounters.

## Introduction

Successful adaptation in sport settings requires effective cognitive, behavioral, and emotional self-regulation skills (Lazarus, 2000b; Tamminen et al., 2014; Crocker et al., 2015). A critical process in self-regulation is coping (Lazarus, 1999), which involves the capacity to modulate thoughts, affects, and behaviors over time and across changing challenging environments (e.g., Gaudreau et al., 2010). Although sport psychology research has primarily focused on coping as a within-person or intrapersonal process, stressful circumstances are often experienced in social groups, as in team sports, and coping emerges as a combination of individual and group efforts (e.g., Lyons et al., 1998; Friesen et al., 2013; Tamminen and Gaudreau, 2014). Successful team adaptation is often the result of teamwork and interpersonal coordination (Friesen et al., 2015). However, to date, little is known about how athletes in a team collectively cope with stressors during competitive sport encounters (e.g., Tamminen and Gaudreau, 2014; Crocker et al., 2015). Thus, the purpose of this study was to extend knowledge on stress and coping processes by exploring shared stressors and the ways in which team sport athletes collectively deal with them by using an interpersonal perspective.

Much of the research on stress and coping within sport psychology is based on Lazarus’s cognitive-motivational-relational theory (CMRT) of emotion (Lazarus, 1999, 2000a). Within this theory, stress is a process that occurs as the result of a transaction, or reciprocal effects, between athletes and their environment (Lazarus, 2000a; Hoar et al., 2010). Sources of stress within these transactions are called stressors and refer to demands that individuals appraise as taxing or exceeding their resources (Crocker et al., 2015). Over the past couple of decades, sport psychology researchers have focused on examining the different events, demands or situations that athletes appraise as stressors (e.g., Noblet and Gifford, 2002; Arnold and Fletcher, 2012; Fletcher et al., 2012). Some of these studies focused specifically on team sports and identified specific stressors appraised by team sport athletes (e.g., Anshel and Wells, 2000; Holt and Hogg, 2002; Noblet and Gifford, 2002; Nicholls et al., 2006, 2009). For example, Anshel and Wells (2000) identified five main categories of stressors experienced by competitive basketball players during a game: interpersonal conflicts, refereeing decisions, personal performance problems, opposition influences, and team behavior. The majority of these studies (e.g., Holt and Hogg, 2002) emphasized in their conclusions that many of the stressors faced by athletes were related to the social interactions in the context of the team environment (e.g., coaches, game demands, getting a bad call by an official). However, a key limitation of these studies is that stressors in team sports have largely been explored from an individualistic or intrapersonal perspective by focusing on how each individual in a team appraises and experiences internal or/and external events as stressors (Tamminen and Gaudreau, 2014).

There is also a clear need to explore how appraisal of stressors by athletes influences and is influenced by other team members during their common unfolding team activity and to what extent sources of stress are shared by teammates during sport encounters (i.e., interpersonal perspective). Lyons et al. (1998, p. 583) proposed defining a communal stressor as “when one or more individuals perceives a stressor as “our” problem (a social appraisal) vs. “my” or “your” problem (an individualistic appraisal).” To date, few studies have adopted an interpersonal perspective and examined to what extent the stressors individually appraised were also appraised and experienced by teammates at the same or different times during specific sport situations (e.g., Tamminen et al., 2016b; Doron and Bourbousson, 2017). Recently, Doron and Bourbousson (2017) investigated the nature of the stressors for nine competitive basketball players on the same team, as appraised during a game, as well as the degree of synchronization of these stressors during an unfolding game. They showed that team sport athletes shared and experienced similar and common game-specific stressors and how such communal stressors are arranged together within the team to generate team level configurations of stress. Their results provided empirical support to research call of Crocker et al. (2015) to investigate how stressors are appraised as a team and how athletes’ shared appraisals may be congruent or incongruent from their teammates’ appraisals. However, the results of Doron and Bourbousson (2017) did not explore the collective efforts teammates made in response to the shared game-specific stressors. Despite the promising results of this study, further information is needed to provide deeper understanding of how athletes interact within the social context of a team to collectively deal with shared stressors (Tamminen and Gaudreau, 2014). Indeed, exploration of how teams can use their collective resources to overcome shared stressors has so far been overlooked (Morgan et al., 2013, 2015; Bowers et al., 2017).

Traditionally, coping is defined as constantly changing “cognitive and behavioral efforts to manage specific external and/or internal demands that are appraised as taxing or exceeding the resources of the person” (Lazarus and Folkman, 1984, p. 141). An athlete’s overall self-regulation repertoire comprises a variety of coping strategies (e.g., effort expenditure, problem-solving, refocusing, seeking social support, relaxation, logical analysis, disengagement, acceptance, venting emotion) that can be grouped into meaningful and parsimonious higher-order dimensions of coping (e.g., Compas et al., 2001). In their recent meta-analysis, Nicholls et al. (2016) proposed a new and comprehensive coping classification: (a) *mastery coping*, including strategies that involve athletes attempting to take control of a stressful situation and thus eliminate the stressor (e.g., task-oriented coping, problem-focused coping, engagement, approach); (b) *internal regulation coping*, in which athletes attempt to manage internal responses to stress (e.g., emotion-focused, acceptance, distraction-oriented); and (c) *goal coping withdrawal*, referring to athletes ceasing their efforts to achieve goals (e.g., disengagement-oriented coping, behavioral disengagement, mental disengagement, venting emotion).

Coping processes, like sources of stress, have been studied almost exclusively from an individualistic perspective (Lyons et al., 1998; Tamminen and Gaudreau, 2014; Crocker et al., 2015). For example, Holt and Hogg (2002) investigated the sources of stress of soccer players and the coping strategies they used to deal with them. While they did not adopt an interpersonal approach in their investigation, they discussed perspectives relating to the importance of training elite team sport athletes in ways of coping with the social stressors associated with their particular team subculture and performance environment; they also discussed developing inter-player communication skills or supportive behavior as team coping strategies. Once again, this study focused on sources of stress and coping strategies in team sports without exploring the collective efforts developed by teammates to deal with common team-specific stressors. Given that team sports denote an inherently social context involving a number of important interpersonal relationships (e.g., between athletes, and between athletes and staff), there is a need for better understanding of coping from a social network or interpersonal perspective (Nicholls and Perry, 2016; Neely et al., 2017).

Communal coping represents a novel perspective through which to examine athletes’ collective efforts to deal with stressors in a team or group context. It refers to a process whereby stressors are appraised and acted upon in the context of close relationships, and describes the efforts of individuals in a group as they collectively cope with stressors (Lyons et al., 1998). To date, this concept has mainly been investigated in the fields of family psychology, health psychology and disaster psychology. Research has focused on circumstances where individuals deal with shared stressful events, such as divorce (e.g., Afifi et al., 2006), health problems (e.g., Rentscher et al., 2015) or natural disasters (e.g., Afifi et al., 2012; Richardson and Maninger, 2016; Wlodarczyk et al., 2016). For example, Wlodarczyk et al. (2016) examined the communal coping strategies of people affected by an earthquake in Chile in 2010. Their results provided evidence that shared stressful life circumstances were a context in which people engage in joint actions and communal coping strategies (e.g., avoiding the situation; regulating emotional responses; redirecting attention) in order to successfully cope with collective trauma. While some initial research has begun to explore interpersonal processes of stress and coping in various aforementioned contexts, studies examining these processes are relatively sparse in sport contexts. However, team sport appears to provide an ideal setting that offers multiple opportunities to provide a deeper understanding of how communal coping operates in various types of teams and in various contexts.

Lyons et al. (1998, p. 592) noted that, “the impact of communal coping is obvious in team sport.” In this field, sport psychology researchers have recognized the potential value of using an interpersonal approach in sport, given the inherently social nature of the activity (Tamminen and Gaudreau, 2014; Crocker et al., 2015). There is some sport related research that is connected to the concept of communal coping. For example, several studies of athletes have shown the benefits of social support in sport (e.g., Holt and Hogg, 2002; Hassell et al., 2010; Tamminen et al., 2016a) or interpersonal emotional regulation (e.g., Friesen et al., 2013; Tamminen and Crocker, 2013; Tamminen et al., 2016b). Recently, team resilience in elite sport has been recognized as an important avenue for researchers to better understand how teams can sustain optimum performance under pressure. New developments in this area of sport psychology research suggest that an understanding of how teams mobilize their collective psychosocial resources to withstand stressors is essential for optimal performance (Morgan et al., 2013, 2015, 2017; Decroos et al., 2017). While this new area of research focuses on the collective resilient characteristics of teams that can protect them from the potential negative effect of the stressors, little attention is paid specifically to the collective efforts and cooperative actions developed by teammates to deal with common team-specific stressors (i.e., communal coping strategies). Nevertheless, recent works have considered the interpersonal processes related to dyadic coping in coach–athlete dyads (e.g., Nicholls and Perry, 2016) or communal coping processes in small groups within families (Neely et al., 2017). For example, Neely et al. (2017) examined how female adolescent athletes and their parents cope with deselection from provincial sport teams using a communal coping perspective. The responsibility for coping with deselection and its consequences appeared to change over time, moving from an “our problem, my (parents’) responsibility” orientation, through “our problem, our responsibility,” to an “our problem, my (athlete’s) responsibility” orientation. Their study contributed to depicting a process of communal coping. Nonetheless, there are no studies to date which have used a communal coping perspective to investigate athletes’ collective coping as a team (Lyons et al., 1998; Tamminen and Gaudreau, 2014; Crocker et al., 2015).

### The Present Study

Considering the widespread use of the CMRT of emotion (Lazarus, 1999, 2000a), it is not altogether surprising that sport researchers have primarily studied stress and coping as an intrapersonal process. Nevertheless, specific coping behaviors of teams might be quite distinct from the individual coping behaviors generally reported by athletes in qualitative studies and through available self-reported questionnaires. On the other hand, communal coping (Lyons et al., 1998) offers a promising perspective to investigate the coping of a team – that is, how the team as a whole copes with the requirements of a collectively shared stressor. However, this concept has mainly been investigated in the fields of family psychology, health psychology and disaster psychology without consensual conceptualization and operationalization of exactly what phenomenon is to be studied. It might be time to envision an extension of stress and coping frameworks in which stress and coping are also operationalized at team level. Given the scarcity of research examining interpersonal processes of stress and coping within teams in sport contexts, qualitative methods were deemed as the most appropriate to explore shared stressors encountered by team sport athletes and communal coping strategies developed to deal with them. The purpose of this study was to investigate stress and coping as an interpersonal process within team sports. Specifically, the communal coping strategies used by the team (e.g., communal actions used by several teammates) to deal with shared stressors were qualitatively explored during competitive encounters.

## Materials and Methods

### Participants

We purposefully sampled French male and female team sport athletes who varied in terms of age (*M*_age_ = 26.3 years, *SD*_age_ = 7.67, range 15–38), gender (three females, seven males), years of practice (*M* = 17.6 years, *SD* = 8.7, range 9–27), levels of practice, and who participated in different team sports. The diversity of the sample allowed us to collect a wide range of information. In addition to these criteria, the 10 participants were selected based on their key roles in their respective teams (see **Table 1**). After eight interviews, when two further interviews had been conducted with no new themes emerging, we defined this as the point of data saturation (Francis et al., 2010). All athletes volunteered to participate in this study. Informed consent was obtained from each player (or their parents in the case of minors) prior to participating. Assurance was given that all information would be confidential and anonymous. Accordingly, participant codes were used in the results section (see **Table 1** for full information). The protocol was also approved by the scientific committee of the French National Association of Research and Technology (ANRT).

**Table 1:** Participant information.

Participant	Age	Years of practice	Sport	Level	Specific role in the team
A1, WFB^a^	25	18	Football	Regional	Former captain
A2, MR^b^	21	8	Rugby	National	Captain
A3, MR^b^	19	6	Rugby	Youth international	Forwards’ leader
A4, MR^b^	22	8	Rugby	International	Kicker and captain
A5, WVB^c^	38	30	Volleyball	National	Captain
A6, MVB^d^	32	18	Volleyball	National	–
A7, MVB^d^	32	17	Volleyball	National	Defensive leader
A8, MIH^e^	32	15	Ice hockey	National	–
A9, WBB^f^	15	6	Basketball	Youth international	Captain
A10, MBB^g^	30	25	Basketball	Regional	–

*^*a*^Women’s football, ^*b*^men’s rugby, ^*c*^women’s volleyball ^*d*^men’s volleyball, ^*e*^men’s ice hockey, ^*f*^women’s basketball, ^*g*^men’s basketball*.

### Procedure and Data Collection

The first author contacted coaches to inform them of the study and asked them to approach athletes. Athletes were then contacted by phone and informed of the specific nature of the study. Data collection involved semi-structured interviews exploring shared game-specific stressors and communal coping strategies in team sports. Semi-structured interviews relied on the CMRT of emotion (Lazarus, 1999, 2000a) and on the Lyons et al. (1998) definitions of shared stressors and communal coping strategies. The semi-structured nature of the interview format allowed for flexibility to explore topics that were personally important to each participant (Patton, 2002).

Two team sport athletes (not among the 10 study participants) participated in first interviews to pilot the interview guide. These pilot interviews allowed the interviewer to become familiar with the interview guide and helped her to find effective ways to help athletes verbalize notions of sharing. For example, it was agreed that athletes should be prompted to explicitly verbalize their team’s coping strategies immediately after the enunciation of each shared stressors, and not to separate the parts of the interview inquiring about shared stressors and communal coping strategies. In addition, for those athletes who had difficulty expressing shared stressors, they have been invited to remind them of recent match scenarios.

The final version of the guide comprised three parts: (a) general information about the athlete’s background (e.g., past sport experience, his/her interest in his/her sport and his/her specific role in his/her team as captain or leader which ensuring a solid understanding of team functioning) and team social context (e.g., relationships between teammates, with the coach); (b) shared sources of stress within the team experienced during competitions (e.g., events which can impact several teammates during the game); and (c) communal coping strategies used by the team to deal with shared stressors (e.g., communal actions used by several teammates). Each athlete was questioned at an interpersonal level on his/her perception of his/her team’s experiences and functioning when faced stressful situations during competitions. General questions were asked to obtain detailed descriptions of team’s experiences and functioning in these situations. For example, athletes were asked, “During a game, what specific incidents or events caused you and your teammates to feel collectively stressed?”, “What disturbed the functioning of your team as a whole?”, and “What were your thoughts and actions immediately after collectively experiencing this unpleasant event?”. In this line, athlete’s use of ‘we-talk’ was thought as an indicator of communal coping and his/her perception that their team collectively deal with stressors (Crocker et al., 2015). Interviews lasted between 39 and 67 min (*M* = 49 min, *SD* = 8.7) and was conducted by the first author, a sport psychologist accustomed to interview techniques. Interviews were audio recorded and transcribed verbatim, resulting in 152 pages of typed data.

### Data Analysis

The research was conducted from a critical realist position, where the reality is assumed to exist but to be only imperfectly apprehendable because of basically flawed human intellectual mechanisms and the fundamentally intractable nature of phenomena (Guba and Lincoln, 1994). Through this ontological position, the analysis was performed following the main principles of the thematic analytic procedure (e.g., Braun and Clarke, 2006; Clarke and Braun, 2016): becoming familiar with the data, generating initial codes, searching for themes, reviewing and refining themes, identifying coherent patterns, defining and naming themes and producing the report. The coding was done by the first author, who had conducted the interviews, and the third author, who had experience in investigating stressors and coping in sport. They inductively coded on a line-by-line basis relevant data related to shared stressors encountered by the team and the communal coping strategies used to deal with them. When athletes reported a stressor as a common stressful event (i.e., not his/her problem or the problem of others, but our problem) (Lyons et al., 1998), the item was coded as a shared stressor (e.g., “*When we see that our best players are beaten, whether it’s in a fight, speed or during play, it’s really difficult for us,”* A8, MIH). In the same way, when athletes reported actions involving the sharing of resources and combining the efforts of several teammates (Lyons et al., 1998), the item was coded as a communal coping strategy (e.g., “*Our common strategy is to ignore*,” A6, MVB). Between each interview coding, the first and third authors shared their coding and discussed their results until agreement was reached.

Then, codes related to shared stressors and communal coping strategies were collated into potential themes, gathering all data relevant to each potential theme. The themes were checked depending on the identification of common features and adaptive functions. For example, communal coping strategies such as analysis and action planning, information sharing, refocusing, going back to basics and effort expenditure were grouped together on the basis that they shared a problem-focused communal effort function. The shared stressors and communal coping strategies’ themes, were labeled using procedures of constant comparison between the coded narratives, the related developed theories and the conceptualizations of the findings. Even though the analysis was inductively conducted, congruence between existing literature on stress and coping and concepts identified in our analysis was examined to label the different codes and themes (Sandelowski, 1993). When proposing new labels for the communal coping strategies and communal coping themes, and when deemed relevant, we tried to get as close as possible to some of the pre-existing labels for individual coping classification. Thus, communal coping themes have been named communal coping dimensions, in agreement with the classic denomination in the literature (i.e., Nicholls et al., 2016). However, this matching procedure was not possible for the stressors’ data because there is no comprehensive classification of stressors in competitive sport contexts (Tamminen and Holt, 2010).

As a final step in the analysis, we tabulated the number of meaning units (MUs) to observe the distribution of stressors and communal coping strategies among the 10 participants. To specify the distribution, coders counted each of the shared stressors and communal coping strategies, and the number of informants that reported each stressor or coping strategy.

### Methodological Rigor

Several precautions were taken to follow a list of criteria such as credibility and critical researcher (Smith and McGannon, 2017). Indeed, the choice of participants with varied experience increased the possibility of shedding light on the research question from a variety of viewpoints (Patton, 1987) and ensured credibility. Secondly, a perpetual agreement was reached among co-researchers and experts. Thus, during the analysis process, the themes were revised several times. The second author played an important role in this phase, serving as a “critical researcher” questioning analytical decisions, possible researcher subjectivities, and offering potential alternative interpretations (Whittemore et al., 2001). Through these exchanges, the initial analysis and classification were discussed, critiqued and repeatedly modified to obtain a version that was satisfactory to the three authors.

## Results

The data analysis procedure revealed 272 MUs comprising 143 MUs related to shared sources of stress and 129 MUs related to communal coping strategies.

### Shared Sources of Stress

The thematic analysis revealed four themes of shared stressors: “Social pressure issues” (35%, *n* = 50), “Relationship between teammates issues” (32.8%, *n* = 47), “Performance issues” (28.7%, *n* = 41), and “Logistical and organizational issues” (3.5%, *n* = 5).

#### Social Pressure Issues

This theme encapsulated the stressors associated with the pressure that the social environment of the team can generate. Social pressure includes behaviors such as an irritating coach, a referee who makes mistakes, an opponent who provokes. This theme also contains all the expectations of outcomes communicated by the media, spectators, leaders, or family. Some athletes, such as A7, MIH, A10, MBB, and A7, MVB cited stressors related to this theme:

A7, MIH: The referee…it’s a big problem because it really throws the team off.
A10, MBB: The coach got mad, he broke his board and got sent off. That doesn’t help us.
A7, MVB: Obviously the stakes influence us, especially when there is a lot of pressure on the matches. Sometimes it helps the team play well, but sometimes it’s a bit inhibiting.

#### Teammate Relationship Issues

This theme included stressors associated with negative teammate behaviors (e.g., repetition of errors, lack of commitment, arrogance) and negative social interactions between teammates. These interactions can be verbal (e.g., a reproach, negative communication) or non-verbal (e.g., aggressive behavior, physical manifestations of disapproval). This theme is illustrated by the following quotes:

A1, WFB: The captain, she didn’t act the way she should, she kept telling us off…and that created conflict between the girls.
A6, MVB: There’s one player in the team who spends all his time waving his arms in the air – to complain – he’s the coach’s favorite, and the public’s too. But I think it bothers the whole team.

Other athletes, such as A3, MR, described a situation in which relationships within their team were threatened by the reflection of one of the teammates:

A3, MR: Let’s take the example of a scrum. If we (the forward) are packed into a scrum and the prop is beaten by his opposite number… it’s difficult for him, it hurts his pride, it’s a big thing. And if, in addition, the number 10 then says to him “he’s getting you sideways, you can’t keep doing that,” he’s going to take it badly. He’s already suffered a scrum, maybe even a penalty, and then, as well as all that, his teammate doesn’t support him and doesn’t try to make him feel any better about it… […] And well, when you’re a forward and you hear that… It’s like you’re having a go at your own team.

#### Team Performance Issues

This theme encapsulated the stressors associated with the team’s performance level. It includes stressors linked to the low controllability of the situation and the score, the domination of the opponents and the decrease in the perception of team-efficacy. A9, WBB, A4, MR, and A8, MIH referred to these stressors in the following terms:

A9, WBB: We were all over the place, the others were counter-attacking all the time.
A4, MR: When you feel you’re not going to make it, even the things you can normally do don’t work. For example, if it’s 2 against 1 and you don’t manage the pass at the right time. Take the Perpignan away match, we knocked on in the in-goal area! At Montpellier, in the first half we were down 30 points to 3… We were being beaten in every area: scrums, passing, kicks, defense… it was a disaster, in the first half we were nowhere!
A8, MIH: When the team is… – traumatized is too strong- but the team is a bit …well, psychologically vulnerable, you really feel it. When it’s tricky psychologically and you’re beaten, you feel it. Everyone feels it. […] When you’re physically swamped by the opposition, that’s the hardest. When we see that our best players are beaten, whether it’s in a fight, speed or during play, it’s really difficult for us.

#### Logistical and Organizational Issues

This theme encapsulated the stressors associated with the organizational aspects of competitions, such as travel or equipment. This theme is illustrated by the following quotes:

A7, MVB: We have a very specific routine on match days, and sometimes, if the journey doesn’t go to plan, we can’t follow it. That can affect us in the evening match.
A5, WVB: We’ve played in some gyms where there were leaks in the roof. Once, someone suggested we play in gloves, it was so cold.

### Communal Coping Strategies

The 129 communal coping strategies used resulted in 13 categories, which then fitted into four overarching communal coping dimensions: “Problem-focused communal efforts”; “Relationship-focused coping”; “Communal management of emotions”; and “Communal goal withdrawal” (see **Table 2**).

**Table 2:** Frequency and intraclass percentage of strategies perceived by team members during a game.

Communal coping dimensions and strategies	*n* (%) participants	*n* (%) occurrences
**Problem-focused communal efforts**	**8 (80%)**	**39 (30%)**
Analysis and action planning	7 (70%)	15 (12%)
Information sharing	7 (70%)	11 (9%)
Refocusing	5 (5%)	5 (4%)
Going back to basics	2 (20%)	4 (3%)
Effort expenditure	3 (30%)	4 (3%)
**Relationship-focused coping**	**10 (100%)**	**38 (29%)**
Motivational support	6 (60%)	19 (15%)
Compensation	6 (60%)	11 (9%)
Social Joining	5 (50%)	8 (6%)
**Communal management of emotions**	**10 (100%)**	**28 (22%)**
Interpersonal emotional regulation	9 (90%)	18 (14%)
Reassurance	4 (40%)	6 (5%)
De-dramatization	3 (30%)	4 (3%)
**Communal goal withdrawal**	**9 (90%)**	**24 (19%)**
Task-disengagement	9 (90%)	16 (12%)
Venting emotions	5 (50%)	8 (6%)

#### Problem-Focused Communal Efforts

This dimension included communal coping strategies aimed at managing and solving problems encountered by several teammates during the competitive event in a collective way (see **Table 2**). Five communal coping strategies were identified in this dimension. (a) Analysis and action planning, consisting of analyzing a problematic situation together and organizing a communal action plan, as shown in the following quotes from two athletes whose teams were then down:

A9, WBB: The strategy is to target the key elements together. Meaning, what we have do to get back into the game. Do we need to be stronger defensively? Do we need to work together more and share the ball around more? Perhaps we need to find a tactical angle?
A10, MBB: We agree what we need to do, and we try to find solutions among ourselves.

(b) Information sharing, consisting of seeking or providing information support to teammates in order to resolve the problem. For example, two athletes said:

A1, WFB: When something like an injury happens I try to give technical advice. […]. I talk to those who were around to give practical advice: “Keep it simple,” “Don’t get worked up,” “If you see it starting to get heated, move away. Until we get a substitution.”
A5, WVB: -*During team out*- We ask the girls on the bench, as they have a different perspective […] we go and ask their advice.

(c) Refocusing, consisting of teammates keeping the team’s attention on task-relevant cues when they are facing a problem, as highlighted below:

A6, MVB: - *After a lost set* – We move on, as if we’re starting a new match. What’s done is done, we focus on what we have to do.
A10, MBB: We could easily have moved the focus away from the referee, saying, “OK, that’s it, we’re going to stop talking about the refereeing now, we’re just going to play.

(d) Going back to basics, consisting of returning to fundamentals, simple game plans, which the team knows inside-out. The following quotes illustrated this strategy:

A5, WVB: We gave a lot in the 1st set. In the 2nd set we started out at the same pace and at a certain moment we decided that maybe we shouldn’t overdo it and we should go back to basics a bit and play a more traditional game.
A8, MIH: Instead of skating about all over the place, we’re going to try and refocus on simple, effective things.

(e) Effort expenditure, consisting of mobilizing the physical and mental resources of the team in order to act together directly on the stressful situation, as highlighted by the following quotes:

A5, WVB: We didn’t even talk. We fought to the very end.
A10, MBB: It means that, on the contrary, we’re going to go all out, we’re going to be accurate in everything we do so that all the players can enjoy playing basketball well! That’s the most important thing!

#### Relationship-Focused Coping

This dimension included coping strategies using cognitive and behavioral efforts to manage and sustain relationships within the team during the stressful episode (see **Table 2**). Three communal coping strategies were identified in this theme: (a) Motivational support, consisting of one or more teammates providing encouragement through behavioral or verbal actions to one or more players in order to sustain the relationship between teammates. For example, two athletes said:

A1, WFB: We gave each other a boost, saying, “Let’s go, team!”
A3, MR: So then we start to win impacts again and move forward, we tell ourselves that they aren’t better than us. Even in scrum phases our teammates encourage us and give us a boost and that helps keep us going.

(b) Compensation, consisting of adapting the team play to relieve a teammate in difficulty and compensate for his or her weaknesses, as shown in the following quotes:

A7, MVB: If a player is struggling, we say “No, don’t worry, we’ll adapt, we’ll make up for it.” We’ll adapt and even if he doesn’t play quite as well or if his distribution of the ball isn’t as good, we’ll make up for all that until he gets back into the game.
A5, WVB: They try to compensate if they can. Certain players try to make up for me so that you can’t actually see that I’m not really in it. But actually if you know where to look, it shows because they don’t compensate very well.
A9, MBB: - *When the point guard has difficulty-* So it means we have to spread the marking out a bit better between the other players.

(c) Social joining, consisting of physically joining forces to deal with the situation. This strategy is illustrated by the following quotes:

A3, MR: We huddle in the in-goal area, for example, when they score a try.
A1, WFB: When we group together, that cuts us off even further from the outside world, in fact it’s a bubble. We’re sort of in a bubble that is the game. We go back into the team bubble.
A9, WBB: For example, when there are free throws, we have what we call “middles”: that’s when we huddle together on the court. The five players get together, and the captain (usually me when I’m not injured) talks to the team.

#### Communal Management of Emotions

This dimension represented communal coping strategies aimed at regulating team emotions generated by the confrontation with shared stressors (see **Table 2**). Three communal coping strategies were identified in this dimension. (a) Interpersonal emotional regulation, consisting of inhibiting one’s own emotions to protect teammates, to regulate a teammate’s emotions or to use the group to regulate one’s own emotions, as highlighted below:

A9, WBB: *-When a teammate gets angry at the referee-* So we had to push her and tell her to calm down. We go over to her and tell her to calm down and to move away from the referee so that he can’t hear her. We do it because we know that when a girl is fixed on a thing like that, she’s going to get angry.
A2, MR: When one guy makes a mistake, I try to calm down the others who want to jump on him.
A3, MR: For touch mistakes, I tend to take the blame so the guys aren’t affected by it.

(b) Reassurance, consisting of helping a teammate in trouble with his/her skill, as highlighted by the following quotes:

A3, MR: *-To a player who has just come into play-* We had to reassure him. Tell him that he knows all the fundamental moves, that he can do it, and it’ll be fine.
A7, MVB: His mates are around and they’re going to try and go over to him and try to reassure him and give him confidence. Later, it might also be up to the coach to do that but it’s the teammates who are on the court. So the first port of call on the court is the rest of the team: we try to reassure him.

(c) De-dramatization, consisting of defusing the situation together, as illustrated by the following quotes:

A7, MVB: The team laughs about the pressure, we make a joke of it. But afterwards, there’s enormous pressure, we make a little bit of a joke of it. We joke about it between partners a bit, we make a joke of it so it’s easier to deal with.
A8, MIH: *-When their goal keeper is continuously angry-* We muck about generally. We defuse the situation.

#### Communal Goal Withdrawal

This dimension represented the strategies through which a team withdraws from the process of actively striving toward the realization of desirable outcomes (see **Table 2**). Two communal coping strategies were identified in this dimension. (a) Task-disengagement, in which teammates collectively cease their efforts to achieve communal goals. For example, two athletes said:

A8, MIH: It was quite clear, they had all given up, the defense effort had just fallen off. We had given up on defensive moves, on challenging shots… and after that, in attack, we’d lost the pace, I mean, we slowed down, we took forced shots.
A8, MIH: Things like that which keep being repeated and show there’s a drop in the team commitment. Yeah, it can happen, a team can just give up.

(b) Venting emotions, in which teammates collectively express and ventilate unpleasant emotional tensions, as highlighted by the following quotes:

A4, MR: I could see the whole team was about to boil over and complain.
A10, MBB: Well, everyone was angry, yeah. Everyone was angry, they were all complaining and saying “It’s rubbish!”.

## Discussion

The purpose of the present study was to explore team sport athletes’ perceptions how their team collectively cope with shared stressors during competitive encounters. This study provides empirical support for recent research perspectives on stress and coping within teams in sport settings (Tamminen and Gaudreau, 2014; Crocker et al., 2015). The findings indicated that team sport athletes appraised several sources of stress as shared stressors or “our problem” (i.e., a social/team appraisal) and engaged in a variety of communal coping strategies to deal with them during competitive encounters.

The team sport athletes experienced numerous stressors during games, which appeared to affect the team’s functioning as a whole. Specifically, the team sport athletes reported communal sources of stress appraised as “our” problem, related to social pressure issues (e.g., opponents’ provocation, coaches’ behaviors), relationships between teammates (e.g., negative behaviors, negative social interactions), performance issues (e.g., low controllability of the score or the situation, domination of the opponents), or logistical and organizational issues (e.g., equipment). Overall, the results complemented findings from previous research using an intrapersonal perspective to explore team sport athletes’ individual sources of stress (e.g., Anshel and Wells, 2000; Holt and Hogg, 2002; Noblet and Gifford, 2002; Nicholls et al., 2006, 2009). In addition, our results supported the notion that stressors commonly identified at individual level also seemed to be identified at team level (i.e., social pressure, relationships between teammates and performance problems) (e.g., Doron and Bourbousson, 2017). As a result, future research on team adaptation could perhaps take into account how stressors are appraised as a team and how athletes’ shared appraisals may be congruent or incongruent from their teammates’ appraisals.

In response to shared stressors, team sport athletes used a variety of communal strategies to deal with them. The communal coping strategies identified can be grouped into four main communal coping dimensions: problem-focused communal efforts (e.g., analysis and action planning, information sharing); relationship-focused coping (e.g., motivational support, social joining); communal management of emotions (e.g., interpersonal emotional regulation, and reassurance); and communal goal withdrawal (e.g., task-disengagement, venting of emotions). The identification of these four coping dimensions at the team level represents an important step in the understanding of communal coping in team sports by offering a new perspective on how teams cope when dealing with communal stressors. Indeed, these communal coping strategies concretely describe the collective actions teammates use to cope with shared stressors (e.g., sharing information and building an action plan together, encouraging each other to remobilise themselves, reassuring a teammate to restore their confidence). While these communal coping strategies share some similarities with individual coping functions (see Nicholls et al., 2016), the originality of our results stems from the identification of the specific forms of coping strategies athletes used at the team level to deal with shared stressors (e.g., Lyons et al., 1998; Tamminen and Gaudreau, 2014; Crocker et al., 2015).

Some of these communal coping strategies identified in the present research have been reported in previous studies conducted on communal coping in different contexts and social groups (e.g., Afifi et al., 2006, 2012; Rentscher et al., 2015; Richardson and Maninger, 2016). For example, problem-focused communal effort strategies, such as information sharing, material assistance, searching for contact, instrumental support or advice (e.g., Richardson and Maninger, 2016; Wlodarczyk et al., 2016) have previously been reported in disaster survivors’ actions to change or resolve stressful situations. Nevertheless, the sport context revealed specific forms of problem-focused communal effort strategies (e.g., refocusing, going back to basics, effort expenditure) depending on the specificity of the sport context which forces its athletes to remain collectively involved in the task to have a chance to perform together. This was also the case for communal goal withdrawal strategies which shared similarities with the avoidance strategies encountered in a natural disasters context but which took a specific form in the sport and performance context. In the context of natural disasters, they represent the survivors’ actions to physically avoid the disaster site or the group’s efforts to act as if nothing had happened (Wlodarczyk et al., 2016), whereas in the sport context, they are more closely associated with goal withdrawal (i.e., ceasing efforts toward goal attainment). While our results appeared to share some similarities with previous studies conducted on communal coping, they also revealed the specific nature of communal coping used within team sports in a performance setting.

Furthermore, our results also revealed one dimension unique to communal coping, which differs from any existing individual coping dimensions (i.e., relationship-focused coping) and has not been previously highlighted at individual level in a sport context. Relationship-focused coping strategies seem to be used in order to maintain and develop the relationship within the team and to obtain collective benefit during the game (Lyons et al., 1998). In the context of relationships between couples, this coping dimension has previously been reported as the set of cognitive and behavioral efforts to manage and sustain social relationships during stressful episodes (e.g., O’Brien et al., 2009). In addition, in natural disaster contexts, Wlodarczyk et al. (2016) reported that social joining coping strategies consisted in helping individuals to build a closer bond when they were dealing with a source of stress. In the present study, relationship-focused coping strategies appeared to be designed to maintain the quality of relationships in the team in order to benefit collective performance during the competitive encounter. Overall, our results revealed how communal coping operates specifically within sport teams in performance settings and provided unique insights into the processes and forms of communal coping in a sport context.

This research is at the early stages of investigation into the interpersonal dimensions of stress and coping in sport and performance contexts (e.g., Nicholls and Perry, 2016; Doron and Bourbousson, 2017; Neely et al., 2017). Although previous research has investigated communal coping processes in a sport context within coach–athlete dyads (e.g., Nicholls and Perry, 2016) or within families (Neely et al., 2017), to date no work has explored these processes within team sports. In addition, in comparison to other fields (e.g., Afifi et al., 2006, 2012; Rentscher et al., 2015; Richardson and Maninger, 2016; Wlodarczyk et al., 2016), it is important to recognize the specificity of communal coping in the particular context of team sport, where performance goals, exceeding limits and confronting adversity are ubiquitous. Thus, team sport athletes use specific communal coping strategies to deal with shared stressors by pooling their resources and developing cooperative actions in an attempt to achieve strong team performances. Qualitative investigation of these communal coping strategies made it possible to identify the concrete means put in place by athletes to cope together. Hence, our results extend the understanding of stress and coping processes as initially defined by the CMRT of emotion (Lazarus, 1999, 2000a) and provide empirical support for the communal coping model (Lyons et al., 1998) in sport contexts.

Our findings also provide a response to researchers who have highlighted the need for further study of how communal coping operates across different types of social groups (e.g., Afifi et al., 2012) and across various contexts (Richardson and Maninger, 2016). More specifically, our findings provide valuable insight into how stressors are approached in team sports and the way in which athletes collaborate to deal with these shared stressors (Tamminen and Gaudreau, 2014; Crocker et al., 2015; Bowers et al., 2017). This study reinforces the importance of using interpersonal approaches when studying the mechanisms of stress and coping in sport, given the inherently social nature of sport (Tamminen and Gaudreau, 2014; Crocker et al., 2015). These results contribute to the field of interpersonal emotional regulation and social support, and represent a first step in taking into account the interpersonal nature of coping in team sport contexts (Holt and Hogg, 2002; Hassell et al., 2010; Friesen et al., 2013; Tamminen and Crocker, 2013; Tamminen et al., 2016b). They also usefully complement studies on team resilience (Morgan et al., 2013, 2015, 2017) by focusing on the specific strategies that teams use when dealing with communal sources of stress. The contribution of these two concepts, communal coping and team resilience, provides a better understanding of how teams mobilize their collective psychosocial resources to withstand stressors and to sustain optimum performance under pressure.

On a practical level, these results reinforce the suggestions made by some authors who have underlined the importance for athletes of coping with social stressors and developing inter-player communication skills or supportive behavior as a team coping strategy (Holt and Hogg, 2002). To date, stress management programs aimed at training in coping strategies have mainly focused on the development of the individual’s coping resources (e.g., McArdle and Moore, 2012). As a result of the present study, it seems important to make athletes and coaches aware of communal coping processes, and to develop and train in effective communal coping strategies to deal collectively with shared stressors. Stress management programs could be developed to help teams (a) collectively resolve problems, (b) strengthen relationships under stressful conditions, and (c) collectively regulate emotions.

Although this study observed a rigorous methodology, some limitations should be raised. The main limitation is that shared stressors and communal coping strategies of team sport were investigated through the perceptions of one member of a team without necessarily taking into account the team as a whole. We made this choice in order to be able to interview athletes from different teams, different sports, of different ages and different levels and thus span a wide range of information without being limited to a single team and its standards. Furthermore, we chose to interview athletes with specific roles (i.e., captain, leader in the field or in the life of the group) that gave him/her a good knowledge of his/her team and that allowed us to have access to a perception of the collective phenomena that could take place within a team. However, this choice has prevented us from verifying with certainty the collective aspect of stressors and the strategies cited. The second limitation is related to the method used (i.e., individual semi-structured interviews). Although this method maximized the ability to extract in-depth information, we were unable to verify the synchronization of the athletes’ responses within their teams. Future research could use Doron and Bourbousson (2017) method or focus group interviews like Morgan et al. (2013) who used this method to develop a definition of team resilience and identified the resilient characteristics with elite sport teams. Thus, the use of focus groups with entire teams or randomly selected team members could lead to insights into coping as a team-regulatory process and overcome these potential biases (Tamminen and Gaudreau, 2014).

## Conclusion

Despite these limitations, this study represents a first step in the study of stress and coping at team level. It highlights the interpersonal nature of the stress and coping processes in team sports. Although this study provides an initial insight, future research could continue the investigation of these collective processes by checking how they are shared, by whom they are initiated and how to train athletes in their use. It could also be useful to examine the antecedents (e.g., team cohesion, role and responsibility in the team or personality of teammates) and the consequences (e.g., team efficacy) of communal coping. Furthermore, it would be interesting to develop specific ways to assess communal coping strategies in sport.

## Ethics Statement

The protocol was approved by the local ethic committee of the French Football Federation and of the two Universities associated to the project. All subjects gave written informed consent in accordance with the Declaration of Helsinki.

## Author Contributions

CL, FD’A-L, and JD conceived the study. CL collected the data and mainly analyzed them with JD. All authors participated in the manuscript redaction. All authors had complete access to the study data that support the publication and approved the manuscript.

## Conflict of Interest Statement

The authors declare that the research was conducted in the absence of any commercial or financial relationships that could be construed as a potential conflict of interest.
